# Management and Prevention of Multidrug-Resistant Bacteria in War Casualties

**DOI:** 10.3390/tropicalmed10050128

**Published:** 2025-05-08

**Authors:** Diana Isabela Costescu Strachinaru, Céline Ragot, Anke Stoefs, Nicolas Donat, Pierre-Michel François, Peter Vanbrabant, Alexia Verroken, Frédéric Janvier, Patrick Soentjens

**Affiliations:** 1 Center for Infectious Diseases, Queen Astrid Military Hospital, 1120 Brussels, Belgium; 2 HOST Iris, 1000 Brussels, Belgium; 3 Department of Microbiology, Percy Military Teaching Hospital, 92140 Clamart, France; 4 Department of Microbiology, Queen Astrid Military Hospital, 1120 Brussels, Belgium; 5 Burn Center, Percy Military Teaching Hospital, 92140 Clamart, France; 6 Val de Grâce Academy, 1 Pl. Alphonse Laveran, 75005 Paris, France; 7 Medical Component Operational Command, Queen Astrid Military Hospital, 1120 Brussels, Belgium; 8 General Internal Medicine, University Hospitals Leuven, 3000 Leuven, Belgium; 9 Department of Microbiology, Cliniques Universitaires Saint-Luc, Université Catholique de Louvain, 1200 Brussels, Belgium; 10 Sainte-Anne Military Teaching Hospital BCRM Toulon, 83800 Toulon, France; 11 Department of Clinical Sciences, Institute of Tropical Medicine, 2000 Antwerp, Belgium

**Keywords:** antimicrobial resistance (AMR), multidrug-resistant organisms (MDRO), war, military, war wound infection, military medicine, war casualty, combat care

## Abstract

The growing threat of antimicrobial resistance (AMR) is a critical issue for both civilians and the military. With each successive conflict, pathogens become more resistant, making the management of infections in casualties increasingly challenging. To better understand the scope and characteristics of conflict-related AMR, a comprehensive literature search was conducted in the PubMed database in April 2025, using defined search terms related to war casualties and antimicrobial resistance. We screened and included 117 relevant publications, comprising original research articles, reviews, case series, case reports, editorials, and commentaries, published in English or French, with no date restriction. This narrative review synthesizes current evidence on multidrug-resistant bacteria most commonly isolated from war casualties, their associated resistance mechanisms, and the microbiological diagnostic tools available at various levels of the military continuum of care (Roles 1–4). It also presents strategies for preventing cross-contamination and infection in resource-limited combat settings and provides practical, field-adapted recommendations for clinicians, from first responders to specialized care providers, aiming to improve infection management in armed conflict zones and mitigate the spread of AMR.

## 1. Introduction

Armed conflicts have been a known driver of antimicrobial resistance (AMR) ever since World War II (WWII), when the widespread use of arsenicals, sulfonamides, and quaternary ammonium compound disinfectants and the introduction of penicillin as both prophylaxis and treatment set the stage for the subsequent emergence of drug resistance [1,2,3,4,5,6]. In modern conflicts, the use of weapons in densely populated urban areas leads to significant numbers of wounded individuals and large displaced populations. This can also result in the collapse of healthcare and sanitation infrastructures, as well as the loss of diagnostic capacities and of healthcare personnel [2,5]. Disruptions to the healthcare system can hinder critical infection prevention efforts and public health initiatives, such as vaccination programs. Additionally, damage to infrastructure providing water and sanitation can accelerate the development of AMR [1,5]. In conflict-affected areas, both injured combatants and civilians are often treated in understaffed and improvised field hospitals, with limited resources, where they are exposed to a wide range of pathogens due to the limited resources available [2]. Key factors contributing to the rise in AMR include mass casualty incidents that overwhelm the emergency services, overcrowding, challenges in enforcing infection prevention and control (IPC) measures, inadequate testing facilities particularly for microbiological and antimicrobial susceptibility testing (AST), the high prevalence of traumatic injuries requiring numerous damage-control surgeries, and the empirical use of broad-spectrum antibiotics [1,7,8]. In recent years, conflict-related heavy metal contamination of the environment has also been suspected of being a possible driver for the emergence of novel mechanisms of resistance [2,9]. Moreover, the AMR problem is not limited to the conflict zones [1,5]. Populations displaced by war flee to other areas or countries, thereby spreading and contributing to the global AMR problem, with far-reaching consequences for healthcare systems everywhere [1,3,5,10]. Additionally, war-inflicted wounds are far more severe and intricate than those seen in civilian medicine. High-velocity projectiles cause significant soft tissue and bone destruction, as well as severe contamination of the wounds. Explosive devices such as bombs, grenades, mines, and improvised explosive devices result in extensive, multi-organ damage due to the blast effect and associated burn wounds [6,7,11]. Colonization and infection with multidrug-resistant organisms (MDROs) can frequently complicate the treatment of war-related injuries, both among civilians and combatants, contributing to increased morbidity and mortality [1,2,6,12,13]. The military literature from the Middle East conflicts of the 1990–2020 period reflect the growing risk of AMR in infections complicating war-related injuries among both military personnel and civilians [2,5,6,8,14,15,16,17,18,19,20,21]. Recent data from the Ukrainian conflict corroborates this trend [10,22,23,24,25,26,27,28,29,30,31,32,33,34,35,36,37,38,39,40,41,42,43,44]. The objective of this study is to provide both civilian and military healthcare professionals caring for war casualties with an up-to-date and practical review regarding the multidrug-resistant (MDR) bacteria most frequently involved in war-related injuries. We will focus on their resistance mechanisms, the types of samples to be collected, the available detection tests and the strategies for preventing cross-contamination and transmission, with particular emphasis on the military setting across North Atlantic Treaty Organization (NATO) Medical Roles 1 to 4, including isolation procedures during medical evacuation (MEDEVAC). Table 1 summarizes the level of care and services provided at each NATO medical support role [45].

## 2. Methods

We conducted a literature search in the PubMed database on 8 October 2024 and again on 16 April 2025, using the terms “Military Wounds” OR “war-related wounds” OR “war wounds infection” OR “war trauma infections” OR “war casualties’ infections” AND “antimicrobial resistance” OR “multidrug resistant organisms” OR “extensively drug-resistant organisms”. We included original articles, review articles, case series, case reports, editorials, and commentaries written in English and French, with no restrictions on the publication date. Correction articles and clinical trial protocols were excluded, and no attempt was made to obtain information from unpublished studies. Only studies concerning bacterial infections were considered; those reporting only fungal infections were excluded.

## 3. Results

We identified 329 articles relevant to our research, of which 117 were considered relevant to our topic.

### 3.1. A Brief History of AMR in War Casualties

During WWII, the widespread use of chemotherapeutic agents, including quaternary ammonium compound disinfectants and antibiotics such as sulfanilamide and penicillin, became commonplace [12]. Topical sulfanilamide was incorporated into the standard treatment of war wounds, and individual soldiers were encouraged to carry it with them and sprinkle it on wounds while awaiting evacuation and medical care. The use of penicillin in the treatment of war traumas started in November 1942, in Oran, Algeria [12,46]. The British began using penicillin topically to prevent infection in war wounds, while the Americans reserved its use for systemic treatment of infected patients [12,46]. As early as 1946, Fleming reported variations in penicillin sensitivity among bacterial species such as Staphylococcus and Clostridium encountered in war wounds and infections [47]. The Korean War (1950–1953) saw the emergence of penicillin and streptomycin-resistant bacteria such as staphylococci and micrococci [12,48]. The standard treatment for wounded US troops in Korea before they were evacuated to the Tokyo Army Hospital included surgical debridement and the use of penicillin with streptomycin. A study of 58 neurosurgical patients from the Tokyo Army Hospital between 1951 and 1952 revealed that 48 of the 58 cases were resistant to penicillin, and 49 of the 58 cases were resistant to streptomycin [48]. Seven cases demonstrated resistance to all agents tested (penicillin, streptomycin, chlortetracycline, oxytetracycline, and chloramphenicol). These patients were infected with *Staphylococcus aureus*, non-hemolytic staphylococci, or mixed flora consisting of combinations of Gram-positive (GP) cocci and Gram-negative (GN) rods [48]. During the Vietnam War (1959–1975), treatment of infected wounds included penicillin combined with a second agent, most commonly streptomycin, followed by chloramphenicol and colistimethate [12]. Kovaric et al. found resistance to the broad-spectrum antibiotics used at the time in all types of GP and GN pathogens recovered in 112 wound cultures from US troops wounded in Vietnam [12,49]. During the Yom Kippur war (1973), there was an increase in carbenicillin-resistant Pseudomonas aeruginosa and gentamicin-resistant *Klebsiella* strains among burn victims treated with gentamicin and carbenicillin [50]. In the early 1990s, methicillin-resistant *Staphylococcus aureus* (MRSA) was a significant concern in both military and civilian burn and trauma centers [6,51]. The Middle East conflicts coincided with a growing AMR problem in military hospitals. A US multicentric study conducted on 2699 medical evacuees following traumatic injury, mostly enlisted personnel from the Afghanistan theater of operations, found a 9.1% incidence of MDR GN bacterial infection [19]. The problem was not limited to military hospitals though. Another Middle East study conducted by the non-governmental organization Doctors Without Borders (Médecins Sans Frontières, Geneva, Switzerland) in the Sulaymaniyah burn center in Iraq between 1 July 2008, and 1 September 2009, found that 92% of GP and 63% of GN isolates from blood cultures were MDROs [20]. Another study, conducted on patients admitted between 2016 and 2019 to the International Committee of the Red Cross (ICRC) reconstructive war surgery center in Dar el Chifaa Hospital, Tripoli, in Lebanon, found a high proportion of MDROs in the samples taken from skin and soft tissues and bones, particularly Enterobacterales (44.6%), MRSA (44.6%), and *P. aeruginosa* (7.6%) [18]. The multivariable analysis indicated that the odds of MDR isolates were higher in Iraqi patients compared to Syrian patients and in Enterobacterales isolates compared to *S. aureus* isolates [18]. A study conducted at the Galilee Medical Center, Nahariya, Israel, on 107 Syrian children, found an MDRO carriage rate of 83%, with extended-spectrum β-lactamase-producing *Enterobacteriaceae* (ESBL), carbapenem-resistant *Enterobacteriaceae* (CRE), and MDR *Acinetobacter baumannii* complex (ABC) being the most frequently isolated MDROs [21]. A systematic review conducted by Granata et al., encompassing 22 studies on patients from the Iraq and Afghanistan conflicts, found an overall MDRO prevalence of 86% among these patients [3]. High MDRO rates were also found in casualties from the Libyan war (2014–2020) [3,52]. A surveillance study conducted in Germany, between August 2016 and January 2017, on 67 military and civilian war casualties injured during the Libyan war, observed that 60% were colonized or infected with MDROs, of which 37.3% exhibited resistance to carbapenems [52]. The most frequent detected carbapenemases in this study were NDM, OXA-48, and OXA-23. The study also identified other β-lactamases with blaCTX-M-group-1 being the most prevalent, and plasmid-mediated quinolone resistance genes (qnrB, aac(6′)Ib-cr) were also identified [52]. A similar worrisome MDRO trend has been observed since the onset of the Ukraine conflict in 2014. Even before the beginning of the war in Ukraine and the arrival of Ukrainian refugees in the European Union, there were reports of transborder spreads of MDROs, such as the spreading of an NDM-1-producing *Klebsiella pneumoniae* ST11 from a victim of the Euromaidan protests in 2014 [22]. A retrospective multi-center microbiological survey was conducted in four Ukrainian military hospitals (NATO Role 3–4) between 2014 and 2020 by Kondratiuk et al., on 162 servicemen. The study found proportions of MDR strains of approximatively 80% among the clinical isolates of *Eschericha coli*, *K. pneumoniae*, and *P. aeruginosa*, and of 60% among *Acinetobacter* species [26]. Following the February 2022 invasion, several European countries received refugees and patients from Ukraine, including civilians and soldiers with war injuries. These countries subsequently noted an increase in MDR carbapenemase-producing Enterobacterales (CPE), including uncommon opportunistic CPE *Proteus* and *Providencia* species and MDR non-fermenters such as *P. aeruginosa* and ABC [27,28,29,30,31,32,33,34,35,36,37,38,39,40,41,42,43]. Schultze et al. found higher prevalences of carbapenem-resistant (CR) bacteria among Ukrainian patients (9.7%) treated in University Hospital Frankfurt am Main, Germany, than previously in refugees from the Middle East who arrived in 2015 and 2016 [28]. CR bacteria were frequent in children and in patients with war-related injuries or after medical pre-treatment in Ukraine. Genetic characterization revealed nine carbapenemase genes, with NDM-1 detected most frequently, and hypervirulence marker genes were present in five of six carbapenem-resistant *Klebsiella pneumoniae* isolates [28]. To date, no study on AMR from the latest Gaza conflict was found, but previous studies showed high rates of MDRO in the region, suggesting an ongoing threat, especially considering the considerable public health setbacks faced by the strip [5,53,54,55].

### 3.2. An Overview of the Most Incriminated Species

#### 3.2.1. *Acinetobacter baumannii* Complex

*Acinetobacter baumannii* complex (ABC) consists of four closely related species that are challenging to differentiate based on phenotypic characteristics. The three most clinically significant species, responsible for the majority of both community-acquired infections (CAI) and hospital-acquired infections (HAI), are *A. baumannii*, *Acinetobacter pittii* (formerly known as *Acinetobacter* genomic species 3), and *Acinetobacter nosocomialis* (formerly *Acinetobacter* genomic species 13TU). The fourth member of ABC is an environmental species, *Acinetobacter calcoaceticus*, which is rarely implicated as a pathogen [56]. ABC are aerobic, pleomorphic GN bacilli that are a well-known cause of HAI and outbreaks, especially in intensive care units (ICU) [57]. They can form biofilms and are intrinsically resistant to desiccation, which contributes to their extended persistence in environments and transmission in healthcare settings [58]. ACB can survive on solid and dry surfaces for months, even years; can evade host immunity; has high levels of innate AMR; and can acquire new antibiotic-resistance mechanisms, making it a redoubtable pathogen [59]. It targets moist tissues such as mucous membranes or injured skin, causing a wide range of conditions such as skin and soft tissue infections (SSTI), urinary tract infections (UTI), pneumonia, meningitis, surgical wound infections, and sepsis [56]. Patients requiring artificial devices such as catheters or ventilators and those who underwent dialysis or antimicrobial therapy within the preceding 90 days were at risk of developing *A. baumannii* infections [58,59]. A study conducted in the Military Medical Academy in Belgrade, Serbia, found higher rates of *A. baumannii* colonization and infection during the June–September 1999 period (corresponding to the NATO bombing of Serbia and the following 3 months), compared to June–September 2000–2004 (peacetime), with transfusion of blood products being the sole independent risk factor for *A. baumannii* infection [60]. ABC gained notoriety during Operation Iraqi Freedom and Operation Enduring Freedom, when it emerged as a significant hospital-acquired pathogen in combat-injured military personnel returning from Iraq, Kuwait, and Afghanistan [3,8,14,15,61,62,63,64,65]. The retrospective study conducted by Keen et al. in a military hospital during the Iraqi conflict saw a significant increase in MDR ABC isolates from 4% to 55% (*p* < 0.009) [65]. Both US and Canadian troops reported outbreaks of MDR *A. baumannii* HAI, especially bloodstream infections (BSI) and ventilator-associated pneumonias (VAP) [61,63]. MDR *Acinetobacter* war wound infections, burn infections, and osteomyelitis were also a problem in military personnel wounded in Iraq [62]. Moreover, in this case series, ABC were resistant to all antimicrobial agents except imipenem and amikacin, making them particularly difficult to treat [62]. High rates of MDR *A. baumannii* were also reported among civilian patients during the Middle Eastern and the Libyan conflict [5,18,20,66,67]. Even more troublingly, the systematic review conducted by Granata et al. found that more than 80% of ABC strains isolated from Middle Eastern patients were MDR [3]. This increase in MDR organisms also translated to novel resistance mechanisms at the molecular level [64]. The ABC isolates were found to carry several carbapenemase genes, such as blaOXA-51-like, blaOXA-23, and blaOXA-69-like, and OXA-58-like [3]. Also, there were reports of transborder spread of carbapenemase genes, such as the detection of blaNDM-1 gene in ABC isolates from four Syrian patients treated in Lebanon [68]. ABC also stood out as an important pathogen in war casualties in the Ukrainian conflict [7,23,24,25,26,40,44]. In the study conducted by Kondratiuc et al., 92.5%, 83.0%, and 67.9% of ABC strains were resistant to fluoroquinolones, aminoglycosides, and carbapenems, respectively [26]. The carbapenem resistance was correlated with the presence of OXA beta-lactamases (blaOXA-23, blaOXA-24, and blaOXA-72) and blaGES-12, as well as various other intrinsic and acquired resistance-associated genetic elements [25,26]. Moreover, there were reports of resistance to last-line antibiotics, such as the series published by Ljungquist et al., where 24% of ABC were cefiderocol-resistant [31]. Considering that carbapenem-resistant ABC infections are the fourth-leading cause of death attributable to AMR worldwide [69], these findings are especially concerning.

#### 3.2.2. *Pseudomonas aeruginosa*

*Pseudomonas aeruginosa* is an environmental GN bacillus intrinsically resistant to a wide array of antibiotics, capable of causing both acute and chronic infections that are associated with high morbidity and mortality in various patient groups [70]. *P. aeruginosa* is a very adaptable pathogen, with a large and variable arsenal of virulence factors and several mechanisms of antibiotic resistance, including multiple chromosomal determinants, as well as complex regulatory pathways involved in intrinsic and adaptive resistance which allow it to escape the host immune response [71]. Moreover, it thrives on wet surfaces and can develop robust biofilms that are highly resistant to antibiotics, disinfectants, and host defenses, impairing bacterial clearance and leading to chronic difficult-to-treat infections, but also making it one of the most redoubted HAI pathogens [70,71]. The role of *P. aeruginosa* in war wound infections has been documented ever since WWII [12]. This was further highlighted during the Vietnam War, when a study conducted on wounded Marines showed that although GP organisms accounted for approximately half of the initial wound isolates, *P. aeruginosa* became the most frequently recovered bacteria by day 5 [13]. As previously mentioned, military personnel deployed in conflict zones are also at risk of burn wounds. In the United States military, approximately 5% of combat injuries sustained during Operation Iraqi Freedom and Operation Enduring Freedom included burn wounds [72,73]. In the systematic review with meta-analysis conducted by Azzopardi et al., *P. aeruginosa* was the most common GN pathogen complicating burn wounds, followed by *Klebsiella pneumoniae* and ABC [74]. This finding was supported by the systematic review by Wild et al., which identified *P. aeruginosa* as the second most frequent pathogen in burn wounds sustained in the Eastern Mediterranean region, after *S. aureus* [6]. This is significant because nowadays burns and multiple shrapnel wounds account for almost 40% of all injuries in armed conflicts [75]. GN wound infections in war casualties, especially those involving MDR strains of *P. aeruginosa*, were also a significant concern during the Afghanistan, Iraq, and Lebanon conflicts, affecting both civilians and combatants [3,6,8,15,18,19,20,72,76,77]. In the study conducted by Ford et al., 10% of *P. aeruginosa* isolates were MDR, associated with prior exposure to antipseudomonal antibiotics (*p* =  0.002) [77]. In the ongoing Ukrainian conflict, *P. aeruginosa* remains a major cause of morbidity, particularly among severely injured combat casualties, with especially concerning rising rates of AMR. For example, Kondratiuk et al. found 80% of MDR strains among the clinical isolates of *P. aeruginosa* from the microbiological survey conducted in four Ukrainian military hospitals between 2014 and 2020 [26]. The isolates had a 55.6% carbapenem resistance rate and contained a wide range of extended-spectrum beta lactamases (ESBLs), carbapenemase genes such as blaNDM-1, blaIMP-1, blaVIM-2, and several genes encoding resistance to aminoglycosides [26]. Even more concerningly, Pallett et al. recently reported 12 cases of MDR *P. aeruginosa* infections in patients with conflict-associated wounds from 3 separate healthcare facilities in Ukraine, 8 of which were producing metallo-β-lactamases [38]. Notably, one isolate co-produced KPC and NDM, another NDM and OXA-48-like, and a third NDM and an imipenemase-type metallo-β-lactamase. Four other isolates produced NDM alone and a fifth one, an imipenemase-type metallo-β-lactamase alone. As seen during the Middle East conflicts, MDR *P. aeruginosa* were also isolated from civilian casualties, with reports of transborder spreading [6,28,31,32]. While, to date, no data are emerging from the current Gaza conflict, previous reports suggested high rates of MDR *P. aeruginosa* in the region [53,54,55].

#### 3.2.3. Enterobacterales

Reports of MDR *Enterobacteriaceae* complicating war wounds started emerging during the Middle East conflicts of the 20th century. During combat operations in Iraq and Afghanistan, extended spectrum β-lactamase (ESBL)-producing *Klebsiella pneumoniae* and *Escherichia coli* were detected in US personnel with infected combat-related injuries [13]. However, the prevalences of MDR Enterobacterales among local wounded civilians were higher than those in combatant ones [16,18,20,78]. A study conducted in a deployed military tertiary care facility in Baghdad, Iraq, during 2003 and 2004, revealed that GP accounted for 71% of isolates from US troops, while cultures obtained from the predominantly Iraqi population found mostly GN bacteria (*K. pneumoniae* represented 13% of isolates). The total number of GP and GN isolates was not reported, but both showed resistance to a broad range of antibiotics [78]. Another study, conducted at the largest US military hospital in Afghanistan on US and Afghan patients from September 2007 through August 2008, revealed that GN bacteria were common among Afghan patients and, of those, 70% were classified as MDR [16]. The most common *Enterobacteriaceae* were *E. coli* (of which 53% were MDR) and *Klebsiella* (63% MDR), and one-half of potential ESBL producers were community acquired [16]. In contrast, the study conducted by Mendes et al. among wounded US military personnel between 2009 and 2015 found a low prevalence of CRE [13]. However, the situation evolved differently in the Ukrainian conflict, where higher rates of CRE were reported both in military personnel and civilian war casualties [3,7,10,25,26,27,30,32,33,37,38,39,40,41,42,43,44]. In a study conducted in four Ukrainian military hospitals between 2014 and 2020 by Kondratiuc et al., aminoglycosides, fluoroquinolones, and third-generation cephalosporins were less effective than carbapenems against *Enterobacteriaceae*, and resistance to carbapenems was 43% in *Escherichia coli* and 33% in *K. pneumoniae*, respectively [26]. *K. pneumoniae* strains carried several resistance genes, including the carbapemenases blaOXA-48 and blaNDM-1, the ESBL genes blaCTX-M-15, blaSHV, blaTEM, and the rmtC 16S methyltransferase [26]. Even more alarmingly, a study conducted on injured Ukrainian soldiers between December 2022 and March 2023 at a hospital in Dnipro, found several *E. coli* and *K. pneumoniae* isolates resistant to imipenem, meropenem, and cefiderocol [30]. Another sentinel study, which tested 154 isolates retrieved from 141 Ukrainian patients (133 adults with war injuries and 8 newborn babies with ventilator-associated pneumonia between February and September 2022), found an overall 58% resistance to meropenem, and extended antimicrobial susceptibility testing of 107 strains revealed 49% resistance to cefiderocol and 9% resistance to colistine [31]. More than 80% of the 45 Enterobacterales isolates in this study were resistant to ceftazidime-avibactam, imipenem-relebactam, and meropenem-vaborbactam, 78% were resistant to cefiderocol, while colistin resistance was comparatively lower at 22% [31]. Of note, *K. pneumoniae* exhibited more than 80% resistance to several β-lactam/β-lactamase inhibitor combinations and to cefiderocol, with nine strains resistant to all antimicrobials tested [31]. After the February 2022 invasion, several countries such as Germany, Denmark, the Netherlands, and Japan reported treating Ukrainian patients colonized or infected with CRE, highlighting the risk of the spread of these challenging organisms [3,7,10,27,28,32,34,35,39,79]. NDM and OXA-48-type genes were the most commonly identified in GN infections related to war wounds from Ukraine and have now become disseminated [3,40]. An estimated 80% of the sequenced *Klebsiella* strains carry the NDM-1 gene—ten times the European average—probably driven by the species’ ability to acquire plasmid-mediated resistance [7]. The situation is increasingly worrisome, with a 2024 study by Ljungquist et al. identifying nine *K. pneumoniae* strains harboring resistance genes to both carbapenems and cephalosporins, as well as *pmrB* and *phoP* mutations linked to colistin resistance [37]. Each colistin-resistant strain also encoded ten virulence factors, indicating pandrug-resistant and hypervirulent phenotypes, with high pathogenic potential [37]. Moreover, multiple types of MDR species or distinct strains of the same species were isolated from same host [39,42,80]. Two such striking cases involved the simultaneous detection of six and eight different MDR strains/species in two individual war trauma victims from Ukraine [39,42]. The coexistence of different resistant species within the same host raises serious concerns, as it facilitates horizontal gene transfer between bacterial species, potentially accelerating the spread of resistance genes from one species to another. MDR *E. coli*, such as the ST-361 strain, carrying multiple carbapenemases and mutations conferring resistance to last-line treatment options, were also increasingly reported [35]. Between May 2023 and January 2024, five carbapenemase-producing *E. coli* ST-361, co-carrying blaNDM-5 and blaKPC-3, which also exhibited reduced cefiderocol susceptibility, were cultured from a war wound of a Ukrainian soldier and peri-rectal swabs of four international soldiers wounded in Ukraine and evacuated to a US military treatment facility in Germany [35]. This is deeply concerning, as *E. coli* is a common commensal bacterium of the human gastrointestinal tract and a frequent cause of both HAI and CAI [81]. Commensal *E. coli* represent a major reservoir for the transmission of antibiotic resistance to other pathogenic bacteria through plasmid exchange and other mobile genetic elements [81]. Therefore, the emergence and spread of such highly resistant *E. coli* strains are a serious public health threat, with possible implications beyond hospital settings, into the broader community. Other less frequent CPE species were also reported. Since March 2022, national surveillance programs in several European countries—including Denmark, Germany, Poland, and the Netherlands—have simultaneously been detecting an increase in opportunistic species such as *Proteus*, *Providencia*, and *Serratia* [3,36,79,82]. To our knowledge, as with *A. baumannii* and *P. aeruginosa*, no data on MDR *Enterobacteriaceae* from the current Gaza conflict have been published. However, previous reports indicated high resistance rates in the region [5,53,54,55,83]. For example, in 2021, Abushomar et al. collected random water and sanitation samples from two major public hospitals (Al-Shifa and the European Gaza Hospital) and found that 34% were contaminated. Among the identified *Enterobacteriaceae*, 22% were ESBL producers, and 11% carried NDM genes conferring carbapenem resistance [83].

#### 3.2.4. Methicillin-Resistant *Staphylococcus Aureus* (MRSA)

The role of *S. aureus* in the infection of war wounds has been documented since WWI, when Whitaker reported 43 *S. aureus* infections in a series of 106 patients having suffered gunshot wounds to the head [84]. Penicillin-resistant *S. aureus* isolates emerged shortly after penicillin was introduced for treatment of bacterial infections during WWII and, within less than two decades, approximately 80% of *S. aureus* strains had developed penicillin resistance, mostly through the acquisition of the *blaZ* gene, which encodes beta-lactamase, an enzyme that inactivates penicillin by hydrolyzing its essential beta-lactam ring [47,85]. This led to the development of semisynthetic penicillinase-resistant beta-lactams, such as methicillin, followed by oxacillin and its derivatives. However, resistance to these drugs developed rapidly as well [85]. Methicillin was approved for clinical practice in 1961, and within the same year, the first clinical isolates of MRSA were identified in a hospital in the United Kingdom [86]. MRSA strains develop resistance to methicillin through horizontal transfer of the *mecA* gene, which encodes PBP2a, a penicillin-binding protein with low affinity for most β-lactam antibiotics. This renders them resistant not only to methicillin, but to almost all β-lactams [85]. Over the following decades, MRSA spread globally, becoming a significant pathogen responsible for hospital-acquired infections in both civilian and military settings. The transmission of MRSA within military environments, particularly in confined spaces such as aboard seagoing vessels or within barracks, poses significant concerns due to the potential for rapid spread [87,88]. One of the earliest documented MRSA outbreaks in a military context occurred in the 1980s at a British Royal Navy hospital [89]. Subsequent reports of MRSA carriage and infections in military settings, both on home soil as well as in deployed operational sites, have persisted over the years [14,88]. In the early 2000s, community-acquired MRSA carrying the Panton–Valentine leukocidin (PVL) gene emerged among US-based military personnel [90]. Colonization with PVL-positive MRSA strains was significantly associated with a higher risk of developing soft-tissue infection. During both Operation Iraqi Freedom and Operation Enduring Freedom, MRSA was consistently one of the top three most frequently identified MDR pathogens [14]. This issue persisted in later Middle East conflicts, impacting not only combatants but also the civilian population. For example, a retrospective cross-sectional study conducted by MSF at an orthopedic center in Iraq found that 81% of 174 patients had at least one MDR organism during their hospital stay, with MRSA being the most common [91]. Similarly, the aforementioned ICRC study found high proportions of MDR pathogens in samples taken from skin, soft tissue, and bone wounds, with MRSA accounting for 44.6% of the cases [18]. Among 67 military and civilian casualties transferred from Libya to German hospitals between 2016 and 2017, 16% were colonized with MRSA [52]. During the Ukrainian conflict, in the study by Kondratiuk et al. on strains collected prior to the 2022 full-scale invasion, GP cocci were generally susceptible to the antibiotics tested; however, 27.3% of *S. aureus* isolates were MRSA. Notably, one of the two *S. aureus* strains studied carried the *mecA* gene, along with the macrolide resistance gene *erm(C)* [26]. More recently, high rates of MRSA carriage were reported among Ukrainian refugees, as documented in a Dutch study [32]. As was the case with MDR GN, we find no available studies from the 2023–2025 Gaza conflict, but previous research showed high carriage rates of MRSA [5,6,55]. For example, in 2022, approximately 65% of *S. aureus* isolates were methicillin resistant [55]. Additionally, *S. aureus* was a major cause of burn wound infections [20,51,92]. Since the 1990s, MRSA has been a major concern in intensive care units treating burn patients and has lately become the leading cause of infection in both civilian and military burn units. [6,20,51,93]. A systematic review by Glas et al., examining pathogens responsible for infections in civilian wounds and burns in conflict-affected countries within the WHO Eastern Mediterranean Region (based on studies published from January 2010 to June 2024), found *S. aureus* to be the most prevalent pathogen (36.3%), with a high proportion of MRSA isolates (55.6%) [6].

### 3.3. Prevention of MDRO Transmission in Military Medicine

Shortly after the start of Operation Iraqi Freedom and Operation Enduring Freedom, a notable rise in infections caused by MDROs, particularly ABC, was observed among wounded US and Canadian soldiers [14,61,62,63,64,65]. Also, several outbreaks of hospital-associated MDR infections were reported among US and Canadian troops during their deployments in Iraq and Afghanistan, initially observed in field hospitals (Role 3) and subsequently spreading to higher-level medical facilities (Role 4), which resulted in increased awareness regarding the importance of infection prevention and control (IPC) measures [61,63]. This led to the adoption of a wide array of IPC interventions aimed at preventing the spread of resistant pathogens [14,61,63,65,94,95]. The US Army Office of The Surgeon General was recommended to train and assign dedicated Infection Control Officers to all deployed hospitals, whose main tasks were to ensure that healthcare providers adhere to essential IPC standards in the combat environment and to oversee the management of the IPC at their respective facilities [94]. Among the responsibilities of the Infection Control Officer were developing standard operating procedures, standardizing clinical practices, ensuring proper patient isolation, and conducting regular monitoring to evaluate adherence to IPC protocols. A specialized 5-day deployment-specific IPC training course was developed for military personnel responsible for performing IPC in the deployed setting [94]. Other interventions that have proven effective within the US military were the development of standardized guidelines, the implementation of rigorous IPC measures both within and outside the combat zone, the isolation of patients evacuated from regions with high prevalences of MDROs from the earliest moments until admission to stateside military healthcare facilities and the active surveillance for MDROs [14,94,95,96]. IPC measures included hand hygiene, contact barrier precautions, patient and staff cohorting, chlorhexidine oral care, and reducing the duration and spectrum of surgical antimicrobial prophylaxis. Implementation of bundles of care to prevent VAP at Combat Support Hospitals in Iraq led to a significant reduction in VAP rates [95].

#### 3.3.1. Recommendations for Isolating Patients

Screening capabilities are usually unavailable in Role 1 and Role 2, occasionally available in Role 3, and always accessible in Role 4 facilities; therefore, preventive isolation should be initiated for casualties injured in regions with a high prevalence of MDROs, such as the Middle East, South Asia, North Africa, and Eastern and Southern Europe. Patients should be placed in contact isolation and screened for MDROs upon admission in the first facility capable of screening. They should remain in isolation until their surveillance cultures return negative results [97,98]. If surveillance cultures are positive, they should be kept in isolation for the entire duration of the hospitalization when possible. If continued isolation is not feasible, it should be maintained until three follow-up cultures taken one-week apart confirm the clearance of the pathogen [97,98,99]. This isolation should begin in Role 3 and continue during the medical evacuation (MEDEVAC) process and throughout repatriation. When the casualty finally arrives to Role 4, he must be suspected of MDRO colonization and appropriate IPC measures must be applied as recommended by national policies. Screening is mandatory and must be repeated before IPC measures are reassessed. These isolation methods are reminiscent of those applied for travelers returning from destinations with high prevalence of MDRO [100,101]. When managing a patient who is potentially or confirmed to be an MDRO carrier, prevention strategies rely on standard precautions, environmental hygiene, adherence to the principle of “clean to dirty” (also known as “advance progression”), the use of personal protective equipment (PPE) by healthcare staff, and, where possible, the use of single-use equipment [97,102,103,104,105].

#### 3.3.2. Advance Progression

To minimize the risk of secondary transmission, patients with MDROs should be placed last in the order of care (for treatments, operations, cleaning, etc.), without compromising their overall care. The benefit–risk ratio should always favor the patient’s safety and well-being [97,102,103,104,105].

#### 3.3.3. Use of Personal Protective Equipment (PPE) and Single-Use Items

To reduce the risk of secondary transmission, healthcare staff must use appropriate PPE when caring for patients with MDROs. This includes gowns, protective aprons, gloves, and goggles in case of fluid projection risk. All used PPE must be discarded within the patient’s room. Healthcare workers exiting the room should be free from any items that may have come into contact with the patient or their immediate environment. PPE should be single-use only. Although this incurs additional costs, it is an effective measure to limit contamination spread. Similarly, equipment that cannot be used once (e.g., blood pressure cuffs, stethoscopes) should be dedicated solely to the patient’s room, cleaned after each use, and left within the room. If feasible, one or more operating rooms should be designated specifically for patients colonized or infected with MDROs [97,102,103,104,105].

#### 3.3.4. Hand Hygiene

Hands are the primary vector for the transmission of MDROs; therefore, hand hygiene is essential in preventing their spread. Handwashing with soap should be performed if hands are visibly soiled or wet. In other cases, the use of alcohol-based hand sanitizers (ABHS) should be prioritized. ABHS are effective against all MDROs and provide complete disinfection, whereas handwashing only reduces bacterial load. The use of ABHS is indispensable for effective infection control [104,105].

#### 3.3.5. Environmental Hygiene

Cleaning surfaces with detergent (soap for example) helps to mechanically remove bacteria present on surfaces. This step is crucial, especially in the presence of visible soiling. The action of the detergent should be followed by disinfection. These bacteria remain susceptible to disinfectants commonly used in healthcare settings, making disinfection an efficient and simple means to limit environmental contamination. Some products combine both detergent and disinfectant properties. A 0.5% bleach solution is an effective, low-cost disinfectant that is easy to implement. It is essential to follow the manufacturer’s recommendations, particularly regarding the required drying time for the disinfectant (which varies depending on the product and concentration), to ensure efficacy [106]. Sanitary facilities require special attention, as they are the most high-risk areas for contamination due to the presence of high quantities of bacteria in stools. In France, the recommendation is to clean and disinfect patient bathrooms twice daily when the patient is suspected or confirmed to be a carrier of MDROs [104,105]. Upon patient discharge, all equipment, including the bed, and the environment should undergo thorough bio-cleaning (several rounds of detergent disinfection, bleach solution, steam treatment, or air-based disinfection procedures) [102,103].

### 3.4. Sampling for MDROs

Rectal screening is the most commonly used method, and often the only one performed [107]. However, it may be accompanied by samples from pharynx, armpit and groin. Nasal screening is typically reserved for the detection of MRSA. The samples must be analyzed promptly (within 24 h). Therefore, it is unnecessary to collect them in the field if no diagnostic tools are readily available and they should only be taken where the appropriate techniques are present. Additionally, it may be necessary to repeat these samples, as the sensitivity is not 100% and initial testing may be negative [108,109]. Moreover, they can become positive later, after antibiotic treatment, due to the selection of the most resistant bacteria.

### 3.5. Detection Methods for MDROs

To date, there is no rapid, simple, or widely accessible method for clinicians to screen for MDROs in Role 1 and Role 2 settings. Identifying these organisms typically requires one of two approaches: microbiological culturing or molecular diagnostic testing from biological samples, both of which are available in Role 3 (infrequently) and Role 4 facilities. Screening for resistance mechanisms usually involves culturing on selective media (which contain antibiotics that inhibit the growth of susceptible bacteria), followed by the generation of an antibiogram to determine the organism’s resistance profile. In recent years, several molecular rapid diagnostic test (RDT) systems have been developed to identify key resistant genes [107]. Some of them are available in clinical practice and may be suitable for use in deployment settings in the near future. However, a key concern is that the fastest diagnostic systems currently available are tested either with colonies that require prior culture-based growth or with basic screening materials, such as swabs from hygiene screenings [88,107]. As a result, further evaluation is needed, as their reliability in diagnosing directly from clinical samples, without prior culture-based growth, is uncertain [88,107]. Molecular techniques, like polymerase chain reaction (PCR), can be used to identify specific resistance genes, such as NDM, VIM, IMP, Oxa48-like, and KPC for CREs, or *mecA* for MRSA. These PCR tests can be conducted on bacterial colonies from cultures, or, in the case of rectal screenings, directly on the biological sample. For CREs, RDTs such as immunochromatographic assays, which are quicker and more cost-effective, have been in use for several years, allowing for the direct detection of carbapenemases from isolated colonies. However, in the case of MDROs, multiple resistance mechanisms may coexist, while molecular RDT systems only detect the more frequent resistance genes [107]. Therefore, they are useful for tracking an outbreak strain with a targeted resistance gene, but not for detecting phenotypic resistance in general. Chromatographic methods provide another diagnostic option, capable of detecting carbapenemase production from either isolated colonies or blood culture bottles. While these tests are more sensitive than previous methods, they do not specify the type of carbapenemase present. For clinicians, understanding the exact resistance mechanism is crucial, as antibiotics effective against one type of carbapenemase may not work against others. Therefore, the antibiogram remains essential for confirming the complete resistance profile and identifying resistance to other antibiotic classes, such as aminoglycosides and fluoroquinolones. However, as mentioned previously, these techniques are not accessible at the Role 1 and Role 2 levels, and even Role 3 facilities often have limited microbiology diagnostic capabilities [110]. Traditional culture-based resistance testing is laborious and difficult to sustain in deployment settings, which makes it seldom available in crisis or war zones, especially in resource-limited environments [5,7,107].

### 3.6. Drawbacks Hampering AMR Mitigation Measures in Conflict Settings

While some IPC measures have proven their value in military settings, particularly during the Middle Eastern conflicts [14,94,95,96], their effectiveness in other war-affected zones is poorly documented. The impact of such interventions in reducing the emergence and propagation of AMR is probably highly context-dependent, with outcomes varying significantly across different regions and different points in time and phases of conflict. During peacetime in high-resource settings, patients colonized with MDROs can often be isolated in private rooms and attended by dedicated care teams, an approach that is largely unfeasible in heavily affected war zones, such as Ukraine and Gaza [5,7]. Overcrowding, power outages, shortages of nursing staff, and the lack of essential consumables such as clean water and alcohol-based disinfectants all pose serious challenges to implementing and sustaining IPC practices [1,5,8]. Surveillance efforts are hindered by limited microbiology capacity, which further restricts the generation of reliable data necessary for AMR mitigation measures [5]. International organizations, such as the WHO and CDC, have provided automated blood culture systems and other diagnostic tools to hospitals in Ukraine. Such efforts are, nevertheless, undermined by the destruction or non-functionality of medical facilities and the need to prioritize acute life-saving interventions [5,7]. In this context, AMR mitigation strategies must be integrated into a broader, long-term humanitarian and public health response that includes both military and civilian healthcare systems, as well as consistent support for infrastructure, training, and essential medical supplies.

### 3.7. Recommendations for Prevention of Combat Injuries Infection

Infections following combat trauma differ massively from those usually seen in civilian settings: often, casualties have deep, penetrating wounds, polytrauma including orthopedic and/or head injuries, burns, and severe blood loss [11,111]. Burn patients are a distinct group of immunocompromised individuals, as burn trauma causes immune dysfunction that affects both cellular and humoral immune systems, while simultaneously triggering a massive systemic inflammatory response [112]. Furthermore, severe burn patients often undergo multiple surgeries, have a higher risk of gastrointestinal translocation, and frequently require ventilation and invasive devices, all of which increase the risk of infection [112]. High numbers of simultaneous traumatic injuries requiring multiple damage-control surgeries, such as mass casualty incidents, can further complicate trauma management in conflict zones. Moreover, treating combat injury infections in a military context is complicated by the continuum of care (Role 1–4). Cleaning and protecting the wounds is essential [12]. The antibiotic prophylaxis varies according to the nation. In the US military, combat medics (military professionals trained in emergency medical care) typically provide first aid in the field (Role 1), and antibiotics are often not administered until patients are evacuated to a battalion aid station (Role 2) for stabilization, before being transported to combat or regional hospitals for more extensive care (Role 3) [111,113,114,115]. When possible, oral antibiotics with broad spectrum activity are preferred for use in combat settings, where the facilities or the environment for intravenous (IV) administration may be lacking [116,117]. When casualties cannot immediately be transferred to a hospital, oral moxifloxacin can be administered to those without penetrating abdominal trauma, shock or needing other oral medication [118]. When oral administration is not possible or in case of penetrating abdominal injuries, US guidelines recommend ertapenem, with cefotetan as alternative, despite its narrower coverage [118]. This recommendation is likely due to its ease of use and reduced logistical complexity, which makes cefotetan more suitable for the battlefield compared to other antibiotics with a spectrum closer to that of ertapenem. After evacuation, the current US guidelines for antibiotic prophylaxis in combat-related injuries recommend the use of intravenous cefazolin for the majority of wounds and levofloxacin or moxifloxacin for penetrating eye injuries [117,118]. Metronidazole is to be added to cefazoline in case of risk of anaerobe contamination, such as perforated digestive tract [118]. Vancomycin is used when there is a suspicion of MRSA [98,100]. In case of delayed evacuation (prolonged casualty care, formerly referred to as prolonged field care), current guidelines recommend a 7- to 10-day course of ertapenem or moxifloxacin, with vancomycin added if MRSA is suspected, for all penetrating trauma [116,118]. The French Armed Forces Health Service recommends amoxicillin–clavulanate for penetrating combat wounds [119]. A single 2 g dose is administered through a direct IV infusion at the Role 1 medical treatment facility, or at the Role 2 facility if Role 1 is unavailable. The association of a single daily dose of aminoglycoside such as gentamycin, infused at Role 2, is recommended in case of type III open fractures (especially type IIIb and IIIc) and abdominal trauma with perforation of hollow viscera [119]. In cases of allergy to beta-lactam antibiotics, amoxicillin–clavulanate is replaced by IV clindamycin in combination with gentamicin. These molecules require slow infusions and are given at Role 2 facilities. The duration of the administration is typically brief, usually extending for 24 h following debridement surgery [119]. There is no evidence that the organisms infecting combat trauma at the time of injury are more virulent or resistant than those associated with civilian trauma [24,116,119,120]. Despite MDROs being a frequent cause of infection in combat casualties in Role 4 facilities, studies have consistently shown that MDRO colonization occurs during hospitalization, rather than through individual carriage or environmental contamination at the time of injury [7,61,76,116,121,122,123,124,125]. This flora, typically acquired 5 to 7 days after injury, is selected by the use of broad-spectrum antibiotics and cross-transmission [122]. Therefore, the current recommendations emphasize using narrow spectrum antibiotics in prevention [118]. Nevertheless, a broader coverage is justified in critically ill casualties with an anticipated delay in evacuation due to the potential for severe consequences from inadequate treatment [116]. After being initially abandoned due to difficulties in application and the introduction of systemic antibiotics that were more effective, easier to use, and safer, recent studies suggest that some topical antibiotics could offer additional value in reducing the risk of infections in combat-related injuries [12,126,127]. Studies have specifically examined the use of topical vancomycin in combat trauma, demonstrating advantages such as MRSA coverage, high antibiotic concentration at the infection site, minimal systemic side effects, and low cost [126,127]. However, correctly applying a powder formulation in combat settings can be challenging, as demonstrated by the difficulties with sulfanilamide during WWII [12]. Also, further research is needed, particularly regarding the risk of selecting GN in the wounds. Other evidence-based measures for preventing infections after combat-related injuries include proper collection and use of cultures, administration of antibiotics within 3 h of injury, initial evaluation by a surgeon within 6 h of injury, early wound cleansing and surgical debridement, frequent changes of wound dressings, use of low-pressure lavage, bone stabilization and management of segmental bone defects, surgical revision of complicated wounds, multidisciplinary guidelines for the treatment of amputees, discontinuation of perioperative antibiotics within 24 to 72 h after surgery, and adherence to IPC measures [96,111,119,128]. Early surgery is a cornerstone of wound management and infection prevention and should therefore be performed as soon as possible, whenever the situation permits. Narrow-spectrum antibiotics should be chosen whenever possible and given for the shortest duration for several reasons: antimicrobial exposure was correlated to colonization by drug-resistant pathogens [129] and the length of antimicrobial therapy was independently linked to the risk of *Clostridioides difficile* infection [130]. Even short courses of broad-spectrum antibiotics had the potential to harm the gut microbiome and increase the likelihood of AMR in non-infectious bacteria, contributing to the further selection and spread of the resistance [131,132]. Notably, even a single dose of antibiotic could disrupt the gut microbiome and promote resistance [133].

## 4. Discussion and Future Directions

The growing AMR threat is a critical issue for the Armed Forces. The nature of warfare is evolving, bringing with it new challenges in the management of combat casualties. Modern conflicts are increasingly occurring in urban, densely populated areas, where the nature of combat greatly alters the way healthcare is provided. The rise of drone warfare, evident in the ongoing conflicts like those in Ukraine and Gaza, is set to render the “golden hour” concept for the management of combat injuries obsolete. This shift may result in prolonged delays for wounded individuals, potentially waiting hours before receiving initial care or being transported to Role 2 medical facilities. The challenges posed by complex trauma and the risk of infection, compounded by the growing prevalence of AMR, particularly to beta-lactams and fluoroquinolones, and increasingly to last-line antibiotics, are significant obstacles to effective treatment. Moreover, in contexts of multiple casualties, overwhelmed medical personnel in front-line hospitals and triage clinics often resort to any available antibiotics to treat the injured, with antibiotic resistance likely a secondary concern [7]. Additionally, disruptions in supply chains force healthcare providers to rely on whatever antibiotics are accessible, resulting in widespread misuse of antibiotics, further exacerbating the AMR issue. This may lead to a scenario where amputation becomes the sole treatment option for MDR combat wound infections. In the interim, prevention remains paramount, and IPC, along with antibiotic stewardship, are crucial in the management of war casualties. In the face of the current AMR crisis, there is an urgent need to support IPC programs in the field and hold all parties accountable for their implementation. This includes enhancing training and promoting a culture of prevention [134]. The impact of IPC in situations where medical evacuation times are prolonged cannot be overstated [134]. Ideally, the military would have broad-spectrum agents that could be used empirically and as early as possible in both combat and healthcare settings to prevent the primary infection, as well as potential nosocomial infections, and reduce the risk of developing sepsis [107]. Topical antiseptics, such as diluted bleach, which is easily available and low-cost, or topical antibiotics such as mafenide acetate, which attains very high local concentrations and is very effective against GN bacteria, are particularly useful for severe, devitalized wounds, or for burns. As the threat of MDROs increases, phage therapy, the use of bacteriophages as a targeted biotherapy against bacteria, could offer either a potential alternative to antibiotics or a complementary method alongside them in the fight against infections [135]. Bacteriophages are viruses that specifically target bacteria without affecting eukaryotic host cells. They can lyse and destroy bacterial cells, making them a promising tool in combating bacterial infections [136]. Phage therapy holds significant potential as a modern military medicine solution, applicable from the battlefield to the home front [135].

## 5. Conclusions

Antibiotic resistance is not only a civilian issue; the military are both contributors and victims. With each successive conflict, managing infections in casualties becomes increasingly challenging due to the rise in antibiotic resistance. Studies have demonstrated that MDRO colonization and subsequent infection are typically hospital-acquired, rather than resulting from individual carriage or environmental contamination at the time of injury. It is imperative to implement IPC measures as soon as practically feasible. Microbiology tools play a pivotal role in both preventing infections and selecting the most suitable treatment. Ideally, these tools should be accessible at least starting from Role 2 (if achievable). Early surgery and administration of appropriate antibiotics are vital. In light of the escalating threat of AMR, effective antibiotic stewardship is paramount.

## Figures and Tables

**Table 1:** Overview of North Atlantic Treaty Organization (NATO) medical support roles *.

Role	Level of Care	Capability
**Role 1**	**Unit-level care (Immediate care, Field level)**: Basic first aid and trauma care provided by unit-level medics or first responders.	Immediate first aid Triage Basic trauma care Stabilization for evacuation to higher roles
**Role 2**	**Advanced medical care**: Care provided at forward operating bases (**e.g., Battalion Aid Station**). Involves stabilization and treatment beyond basic care.	Advanced trauma management Initial resuscitation Limited diagnostic and surgical capabilities (basic surgery for non-complicated injuries) Care for moderate trauma cases requiring immediate attention Administration of antibiotics Stabilization for evacuation to higher roles
**Role 3**	**Field or regional hospitals**: Comprehensive medical care provided at rear operational areas with better facilities.	Capabilities to handle serious combat injuries (extensive wound care and trauma surgery) Intensive care Blood transfusions Advanced diagnostic capabilities Specialist consultations Stabilization for evacuation to Role 4
**Role 4**	**Hospital care (e.g., Military Hospitals, Civilian Hospitals)**: Full range of medical services provided in a hospital environment.	Advanced medical treatment and diagnostics Major surgeries and specialized care Long-term care, rehabilitation and reintegration into military or civilian life Care for non-combat-related injuries or conditions

* These are the minimal required standards of medical support. NATO members are free to upgrade them.
